# PPAR γ Prevents Neuropathic Pain by Down-Regulating CX3CR1 and Attenuating M1 Activation of Microglia in the Spinal Cord of Rats Using a Sciatic Chronic Constriction Injury Model

**DOI:** 10.3389/fnins.2021.620525

**Published:** 2021-03-24

**Authors:** Xilei Li, Qulian Guo, Zhi Ye, E. Wang, Wangyuan Zou, Zhihua Sun, Zhenghua He, Tao Zhong, Yingqi Weng, Yundan Pan

**Affiliations:** ^1^Department of Anesthesiology, Xiangya Hospital of Central South University, Changsha, China; ^2^National Clinical Research Center for Geriatric Disorders, Central South University, Changsha, China

**Keywords:** PPAR γ, microglia, neuropathic pain, CX3CR1, neuroinflammation

## Abstract

**Background:**

Previous studies have proved that peripheral nerve injury is involved in the pathogenesis of neuropathic pain (NP). The peripheral nerve injury primes spinal M1 microglia phenotype and produces pro-inflammatory cytokines, which are responsible for neurotoxic and neuronal hyper-excitable outcomes. Spinal peroxisome proliferator-activated receptor gamma (PPAR γ) has been shown to play an anti-inflammatory role in the development of NP. However, the role of PPAR γ in attenuating the pathological pathway of spinal microgliosis is still unknown.

**Methods:**

Sprague-Dawley rats (male, aged 8–10 weeks) were randomly divided into three groups, i.e., a control group, a NP group, and a NP + lentivirus encoding PPAR γ (LV-PPAR γ) group. The sciatic chronic constriction injury (CCI) model was used to induce NP in rats. Pain behavior was assessed by monitoring the rat hind-paw withdrawal threshold to mechanical stimuli and withdrawal latency to radiant heat. The LV-PPAR γ was intrathecally infused 1 day before CCI. Western blot analysis and real-time qPCR were used to detect the microglia phenotypic molecules and CX3CR1 expression in the spinal cord. *In vitro*, BV-2 microglia cells were transfected with LV-PPAR γ and incubated with lipopolysaccharides (LPS), and the levels of M1 microglia phenotypic molecules and CX3CR1 in BV-2 microglia cells were assessed by western blot analysis, real-time qPCR, and enzyme-linked immunosorbent assay.

**Results:**

Preoperative intrathecal infusion of LV-PPAR γ attenuated pain in rats 7 days post-CCI. The M1-microglia marker, CX3CR1, and pro-inflammatory signaling factors were increased in the spinal cord of CCI rats, while the preoperative intrathecal infusion of LV-PPAR γ attenuated these changes and increased the expression of IL-10. *In vitro*, the overexpression of PPAR γ in BV-2 cells reduced LPS-induced M1 microglia polarization and the levels of CX3CR1 and pro-inflammatory cytokines.

**Conclusion:**

Intrathecal infusion of LV-PPAR γ exerts a protective effect on the development of NP induced by CCI in rats. The overexpression of PPAR γ may produce both analgesic and anti-inflammatory effects due to inhibition of the M1 phenotype and CX3CR1 signaling pathway in spinal microglia.

## Introduction

Neuropathic pain (NP) following an injury to the nervous system is characterized by sustained allodynia and hyperalgesia. NP presents a challenging medical problem because it is refractory to current treatments. Neuroinflammation caused by an activated spinal microgliosis has been considered one of the prominent pathological mechanisms of NP (Calvo and Bennett, 2012; Chen et al., 2018; Shen et al., 2019).

In the spinal cord, microglia respond to a peripheral nerve injury by producing inflammatory mediators, which lead to neuronal hyper-excitability and central sensitization (Gu et al., 2016; Donatien et al., 2018; Shen et al., 2020). The reactive microglia participating in the neuroinflammation are classified into two main states of activation: the M1 phenotype characterized by secretion of pro-inflammatory factors, and the alternative anti-inflammatory M2 phenotype involved in the resolution of inflammation. It is clear now that the M1 microglia phenotype and its cytokines, such as tumor necrosis factor-alpha (TNF-α) and interleukin-1 beta (IL-1β), cause prolonged neuroinflammation and pain pathology in the central nervous system (CNS) (Xu et al., 2016; Lee et al., 2018).

A chemokine receptor located on the microglia in the nervous system, namely CX3CR1, is proven to be involved in neuroinflammation. Compelling pieces of evidence have revealed that the signaling of CX3CR1 in the spinal cord is involved in pain pathogenesis (Xu et al., 2016; Zhang et al., 2017). The levels of CX3CR1 are increased in the spinal cord of rats suffering from tetanic sciatic stimulation-induced NP or complete Freund’s adjuvant-induced inflammatory pain (Wang Z. C. et al., 2018; Li et al., 2019). Additionally, the knockout of CX3CR1 in mice attenuated the nociceptive response in the NP model of partial sciatic nerve ligation (Staniland et al., 2010). Recent studies have shown that the M1 phenotype of microglia involving the upregulation of CX3CR1 and the activation of intracellular p38MAPK and ERK signaling pathways play a pivotal role in the production of pro-inflammatory cytokines. This imbalance between pro-inflammation and anti-inflammation in the spinal cord causes NP (Wang Z. C. et al., 2018; Zhong et al., 2018).

Peroxisome proliferator-activated receptor gamma (PPAR γ), a member of a large group of nuclear receptors controlling the proliferation of peroxisomes, has been shown to have an anti-inflammatory function in several types of macrophagous cells (Jiang et al., 1998; Ricote et al., 1998;

Pascual and Glass, 2006). Studies have demonstrated that the PPAR γ located at the spinal cord is implicated in pain processing (Kondo et al., 2018; Zhong et al., 2019). Based on animal studies, PPAR γ is identified as a potential therapeutic target for NP and its agonists offer protective effects on the development of nociceptive behaviors in a microglial-dependent manner (Maiko et al., 2013; Kondo et al., 2018). Even though it is suggested that PPAR γ signaling may modulate the conversion between microglial phenotypes, its effects on the regulation of microglia-derived inflammatory pathways remain to be demonstrated. Analgesia produced by PPAR γ may involve suppression of intracellular pro-inflammatory signaling pathways, such as nuclear factor kappa B, and modulation of other anti-inflammatory mediators, such as resolvin D1 (Saito et al., 2015; Ruiz-Miyazawa et al., 2018).

In this study, the role of intrathecal infusion of lentivirus-encoding PPAR γ for preventing NP in a rat model of sciatic chronic constriction injury (CCI) was examined along with the *in vitro* effects of PPAR γ on M1 microglia activation in BV-2 cells. The study aimed to explore the effects of PPAR γ on NP via regulating CX3CR1 expression, p38MAPK and ERK signaling, and M1 microglia-derived neuroinflammation in the spinal cord. These findings may help elucidate the role of PPAR γ in pain management, thereby providing novel theoretical guidance for the therapy of NP.

## Materials and Methods

### Animals

All experiments were approved by the Institutional Animal Care and Use Committee of Central South University and carried out following the ethical guidelines of the International Association for the Study of Pain (Zimmermann, 1983; Ogden et al., 2016). Male Sprague-Dawley rats (8–10 weeks old) weighing 250–300 g were purchased from the Laboratory Animal Center of Xiangya Hospital, Central South University. All rats were housed in a specific pathogen-free environment with 12 h light-dark cycles and fed with a rodent diet and water. Animals were randomly assigned to the treatment or control group and allowed to adapt to these conditions for at least 5 days before procedures. All rats were anesthetized with 2–3% isoflurane. Animals that showed motor weakness or signs of paresis after procedures were euthanatized with carbon dioxide and excluded from the study.

### Intrathecal Administration of Lentivirus Vector

For intrathecal injection, a catheter was implanted according to a method described previously (Pan et al., 2010). Briefly, rats were anesthetized and a partial laminectomy at L5/6 was performed to expose the dural membrane. A sterile PE-10 catheter was inserted through a dural incision and its tip was placed at the level of spinal lumbar enlargement. The catheter was secured with 4/0 silk threads to the muscles. After the procedure, all rats were allowed to recover for a week before the CCI surgery. Proper location of the catheter was verified through hind limb paralysis after intrathecal injection of 10 μL of 2% lidocaine.

A cDNA fragment containing the entire coding sequence of rat PPAR γ gene (No. NM_013124), which was marked by enhanced green fluorescence protein (EGFP), was ligated into a lentiviral vector. The recombinant lentiviral vector encoding PPAR γ was constructed and purchased from GeneChem (Shanghai, China). Recombinant lentiviral vector (5 × 10^8^ transducing units/mL) encoding PPAR γ (LV-PPAR γ) or negative control sequence (LV-con) was administered in a volume of 15 μL followed by a flush of 5 μL of saline to ensure the vector was delivered into the subarachnoid space. The lentiviral vectors were injected intrathecally 1 day before the CCI surgery.

### Neuropathic Pain Model

In accordance with the previous studies (Pan et al., 2010; Wang et al., 2019), sciatic CCI was induced in rats. Briefly, the rats were anesthetized and the sciatic nerve at the mid-thigh level on the left side was exposed. The four snug ligatures of the chromic gut suture were loosely tied around the sciatic nerve with about 1 mm space between the knots above its trifurcation. In the sham group, the left sciatic nerve of the rats was exposed without ligation.

### Behavior Tests

The rats were allowed to acclimate to the testing environment for 30 min before assessing the baseline pain threshold. The pain threshold of rats was tested 1 day before CCI surgery (baseline) and once daily on each postoperative day. Each trial was repeated 3 times with an interval of approximately 5 min, and the mean value was calculated. All the behavioral tests were performed between 10 AM and 3 PM by an examiner blinded to the treatment groups.

The mechanical withdrawal threshold (MWT) test was used to assess mechanical allodynia. The hind paw withdrawal threshold (PWT) was measured at the left hind paw of rats using responses to stimulation of von Frey filaments according to the previously described “up-down” method (Chaplan et al., 1994). A series of von Frey filaments (0.6–26.0 g) (Stoelting, Wood Dale, Italy) with increasing stiffness were applied on the central plantar surface of the left hind paw. The filament was bent for 5 s with a sufficient force and a positive response was noted if the hind paw was sharply withdrawn. The PWT was determined by the lowest force evoking three positive responses out of five stimulations. The test was repeated five times, and the average value of the 50% withdrawal threshold was calculated.

Paw withdrawal latency (PWL) in response to noxious radiant heat was used to evaluate thermal hyperalgesia. The latency of paw withdrawal to a noxious heat stimulus was measured by the use of a Hargreaves apparatus (Ugo Basile, Comerio, Italy) according to the previously described method (Pan et al., 2010). Rats were placed separately on a temperature-controlled, 3-mm thick glass floor under which a lightbox was located. The movable radiant-heat source beneath the glass floor was focused on the plantar surface of the hind paw, a withdrawal or flick of the paw was observed and the PWL was recorded on the screen simultaneously. Light intensity was preset to obtain a baseline PWL of approximately 12 s and a cutoff was set at 20 s to avoid tissue damage.

### Cell Culture

BV-2 microglial cells were obtained from a German collection of microorganisms (Deutsche Sammlung von Mikroorganismen und Zellkulturen). BV-2 cells were grown and maintained at 37°C in a 5% CO_2_ atmosphere in Dulbecco’s Modified Eagle’s Medium containing 10% fetal bovine serum, 1% penicillin, and 1% streptomycin.

### PPAR γ Transfection and Lipopolysaccharide Treatment

Cells were plated at a concentration of 1 × 10^5^ cells/well in 6-well plates and transfected with the lentiviral vector in the medium, and the required lentivirus was at a multiplicity of infection of 50 plaque-forming units/cell. After 12 h, the primitive medium was replaced with a medium containing 10% FBS. The experiments were performed on day 3 after transfection. The transfection efficiency was monitored with EGFP. Five visual fields were randomly chosen to count the percentage of EGFP-expression cells by comparing cells in bright and fluorescent fields with a microscope. Cells with stable expression of EGFP were harvested for further experiments.

Lipopolysaccharides (LPS) was applied to cells at a final concentration of 1 μg/mL for the indicated time. A serum-free medium was used as the negative control. After the treatment, the supernatants and cells were harvested for subsequent analysis.

### Western Blot Analysis

Spinal cord tissues or BV-2 cells were collected and treated with the lysis buffer (Solarbio, Inc., Beijing, China) and then mechanically digested to release the proteins. The lysate was centrifuged at 12,000 rpm for 20 min and the supernatant was taken for Western blot analysis. Protein concentration in cell lysates was determined using a protein assay kit (Bio-Rad, Hercules, California). An equal amount of protein (30 μg) was loaded on each lane, separated by 10% sodium dodecyl sulfate-polyacrylamide gel electrophoresis, and transferred onto polyvinylidene difluoride membranes (Merck Millipore). The membranes were blocked with 5% non-fat milk and 0.2% Tween 20 for 1 h at room temperature (RT) and incubated overnight at 4°C with primary antibodies. Subsequently, the blots were incubated for 2 h at RT with horseradish peroxidase-conjugated secondary antibodies. The primary and secondary antibodies used for the Western blot analysis are listed in Table 1. The bands were visualized using enhanced chemiluminescence (Merck Millipore) and exposed onto X-ray films for 1 to 10 min. All Western blotting experiments were performed at least three times to obtain parallel results. X-ray films with blotting bands for each sample were scanned and analyzed with Image Lab 3.0 software (Bio-Rad). The quantitative measurement of protein was done by densitometric analysis and expressed as a relative densitometric unit to that of the control protein.

**TABLE 1:** List of primary and secondary antibodies used for western blot analysis.

Antibody	Company	Catalog number	Dilution
Integrin αM	Santa Cruz	sc-6614	1:100
CX3CR1	Merck Millipore	AB1892	1:1000
p-p38MAPK	Cell signaling technology	4511S	1:1000
p-ERK	Cell signaling technology	4370	1:1000
IL-10	Santa Cruz	sc-1783	1:100
PPARγ	Santa Cruz	sc-7196	1:200
CD86	Omnimabs	OM274900	1:200
β-actin	Proteintech	60008-1-Ig	1:5000
Anti-Rabbit IgG	Merck Millipore	AP132P	1:3000
Anti-mouse IgG	Merck Millipore	AP124P	1:3000
Anti-goat IgG	Proteintech	SA00001-3	1:3000

### Real-Time qPCR

Total ribonucleic acid (RNA) was extracted from spinal cord tissues or cells with a Total RNA Kit (Omega bio-tech) and reversely transcribed to the complementary DNA (cDNA) using an oligodeoxythymidylic acid primer and a TransScript All-in-One First-Strand cDNA Synthesis SuperMix (Transgen Biotech) according to the manufacturer’s protocol. The real-time polymerase chain reaction was carried out using cDNA and All-in-One qPCR Mix (Gene Copoei) on an ABI PRISM7000 machine (Applied Biosystems). PCR was performed at 95°C for 10 min, followed by 40 cycles of 94°C for 30 s, then 59.8°C for 30 s and 72°C for 24 s. The primers were designed using Primer Express software (Applied Biosystems). β-actin gene expression was used as an internal control. The sequences of the forward and reverse primers are shown in Table 2. The relative mRNA expression levels of target genes were measured using the comparative threshold cycle (Ct) and calculated by the 2^–ΔΔCt^ method (Pfaffl, 2001).

**TABLE 2:** Primer set list for real-time qPCR.

**Target gene**		**Primer sequence 5′-3′**
CX3CR1	Forward	GGAGACTGGAGCCAACAGAG
	Reverse	TCTTGTCTGGCTGTGTCCTG
IL-1β	Forward	AGTCTGCACAGTTCCCCAAC
	Reverse	TTAGGAAGACACGGGTTCCA
TNF-α	Forward	GATTATGGCTCAGGGTCCAA
	Reverse	CTCCCTTTGCAGAACTCAGG
PPARγ	Forward	GCCAGTTTCGATCCGTAGAA
	Reverse	AATCCTTGGCCCTCTGAGAT
β-actin	Forward	TCGTGCGTGACATTAAAGAG
	Reverse	ATTGCCGATAGTGATGACCT
CD86 (*in vivo*)	Forward	CTCAGTGATCGCCAACTTCA
	Reverse	ATCTGCATGTTGTCGCCATA
CD86 (*in vitro*)	Forward	GAAAGAGGAGCAAGCAGACG
	Reverse	TCTCCACGGAAACAGCATCT

### Immunocytochemistry

Cells were fixed with 4% paraformaldehyde for 15 min at RT and washed three times with phosphate-buffered saline. Then, the cells were incubated with 5% horse serum albumin in phosphate-buffered saline containing 0.1% Trion X-100 at RT for 30 min to block the unspecific binding of antibodies. After the blocking solution had been washed out, cells were incubated overnight at 4°C with rabbit anti-PPAR γ (1:100, sc-7196; Santa Cruz). Then, the cells were washed and incubated for 2 h at RT with Alexa Fluor^®^ 555-conjugated goat anti-rabbit immunoglobulin G (1:1000, A-21434; Invitrogen) and the nuclei were stained with 4′,6-diamidino-2-phenylindole (DAPI) (1 μg/mL, D9542; Sigma-Aldrich) for 10 min. Fluorescent images were captured with a fluorescence microscope (LSM 510 META; Carl Zeiss, Stuttgart, Germany).

### Enzyme-Linked Immunosorbent Assay

The supernatants of cultured BV-2 microglial cells were collected, and levels of IL-1β and TNF-α were detected by Enzyme-Linked Immunosorbent Assay (ELISA) kits (CUSABIO) according to the manufacturer’s instructions. The concentrations of IL-1β and TNF-α were calculated according to a standard curve generated using the standard substance in the kits.

### Cell Viability Test

Cell counting kit-8 (CCK-8) assay (CK04, Dojindo, Japan) was used to detect the viability of cells. Cells were seeded in triplicate at a density of 1 × 10^5^ cells/well on a 96-well plate and treated with lentivirus transfection or LPS (1 μg/mL) for 72 h. About 10 μL of CCK-8 reagents were added into each well and the cells were incubated at 37°C for 2 h. Each sample was measured at a wavelength of 450nm to determine its absorbance value. Cell viability was calculated by the formula: Cell viability (%) = 100% – (absorbance value of the experimental group – absorbance value of the control group)/(absorbance value of the control group – absorbance value of the blank group).

### Statistical Analysis

All data were presented as mean ± standard error of mean (SEM), and the experiments were performed in triplicate. Data analysis was performed using GraphPad Prism 5 software (GraphPad software, Inc., La Jolla, CA, United States). One-way ANOVA with *post hoc Tukey* test was used to compare the Western blot and qPCR data of different experimental groups. Two-way ANOVA followed by Bonferroni’s *post hoc* tests was used to analyze the behavioral data between different groups. Differences between the two groups were analyzed using Student’s *t*-test. A value of *p* < 0.05 was considered statistically significant.

## Results

### Effects of PPAR γ on the Development of Neuropathic Pain in CCI Rats

**FIGURE 1:**
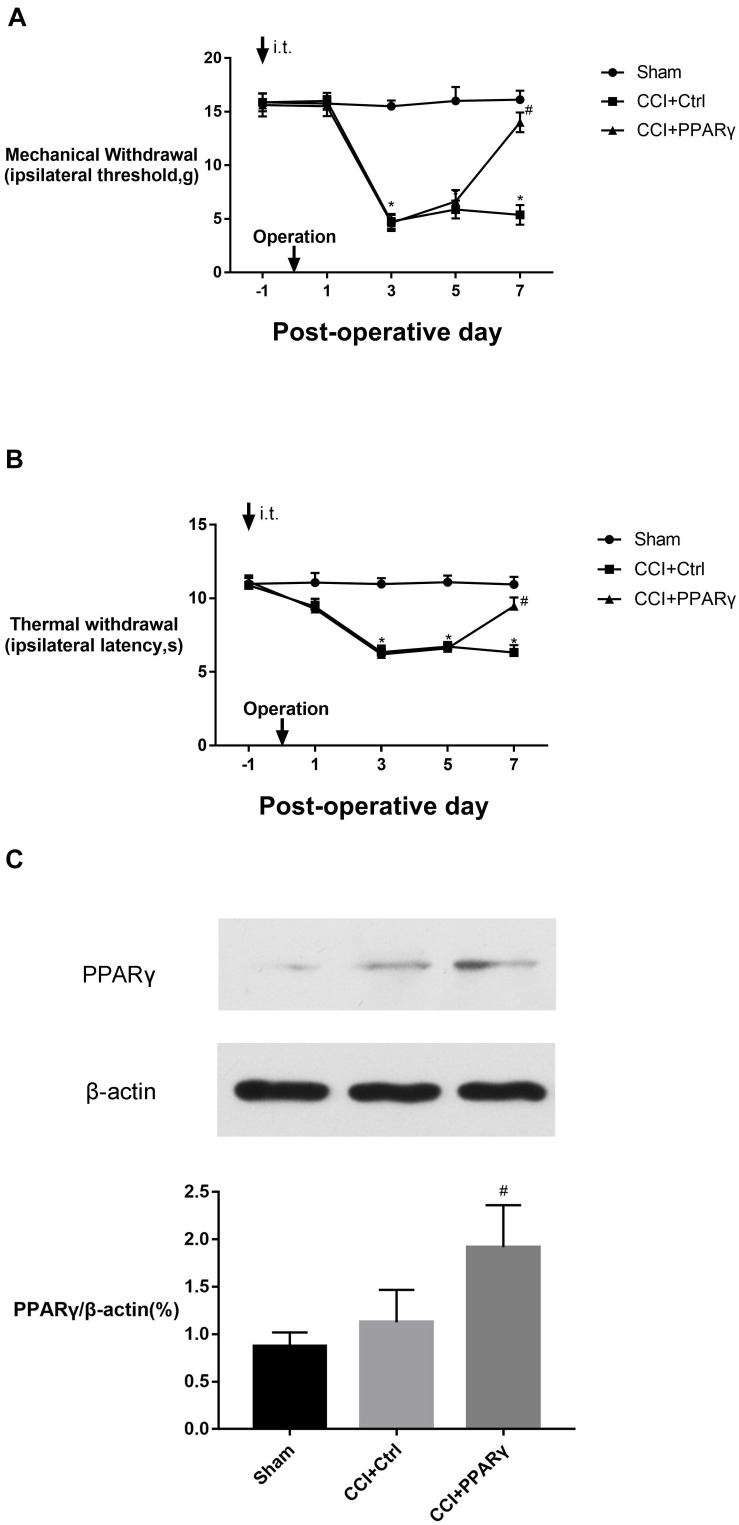
Effects of intrathecal (i.t.) injection of LV-PPAR γ on pain behaviors in CCI-induced rats. **(A,B)** The ipsilateral hind paw withdrawal threshold **(A)** and paw withdrawal latency **(B)** decreased in the CCI group on postoperative days (POD) 3 to 7, compared to the sham group. On POD 7, pain thresholds increased in the CCI + LV-PPAR γ group compared to the CCI group. Arrows indicate the time of intrathecal injection and CCI operation. **(C)** Western blotting showed that the expression of spinal PPAR γ was increased in the CCI + LV-PPAR γ group on POD 7 compared to the CCI group. The results of pain threshold are expressed as mean ± SEM, *n* = 8; the results of PPAR γ expression are expressed as mean ± SEM, *n* = 4. *P < 0.05 vs. sham group; ^#^*P* < 0.05 vs. CCI + control group.

Figures 1A,B showed that the MWTs and PWLs in CCI rats were significantly decreased on postoperative days (POD) 3 to 7 (*p* < 0.05), indicating the NP model was successfully established. To investigate the effects of PPAR γ on NP, intrathecal injection of LV-PPAR γ was administrated 1 day before CCI surgery, and spinal PPAR γ expression was examined in rats on POD 7. Figure 1C showed that PPAR γ protein expression increased in the CCI + PPAR γ group compared to CCI groups (*p* < 0.05), suggesting that spinal PPAR γ was overexpressed in rats after intrathecal injection of LV- PPAR γ. Figures 1A,B showed that MWTs and PWLs were increased in the CCI + PPAR γ group on POD 7 compared to CCI groups (*p* < 0.05), indicating that spinal PPAR γ up-regulation prevented the development of NP.

### Effects of PPAR γ on M1 Microglia Activation and the Expression of CX3CR1, IL-10, p38MAPK, and ERK in Spinal Cord

**FIGURE 2:**
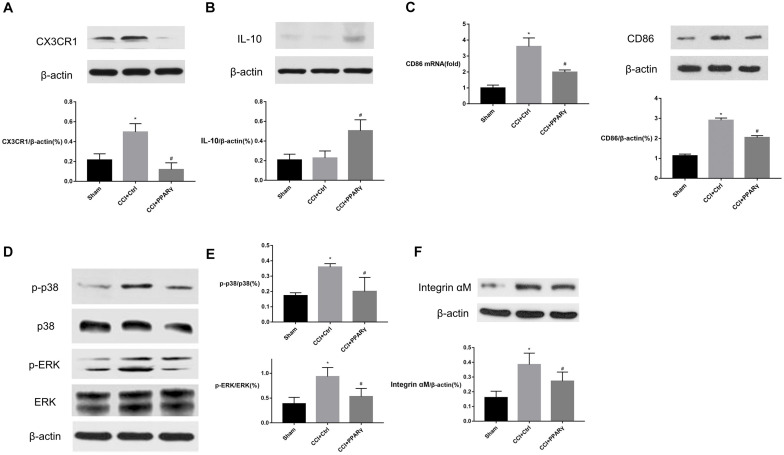
Effects of PPAR γ on the expression of CX3CR1 **(A)**, IL-10 **(B)**, p38MAPK and ERK **(D,E)**, and microglia activation **(C,F)** in the spinal cord of CCI rats. **(A–F)** Western blotting analysis showed that the levels of CX3CR1 **(A)**, phosphorylated p38MAPK and ERK **(D,E)**, and Integrin αM **(F)** were significantly decreased, and the level of IL-10 **(B)** was significantly increased in the CCI + PPAR γ group compared to the CCI rats. Quantification analysis of real-time qPCR and western blotting analysis showed that the levels of CD86 mRNA and protein **(C)** were decreased in the CCI + PPAR γ group compared to the CCI rats. The results are expressed as mean ± SEM, *n* = 4. *P < 0.05 vs. sham group; ^#^*P* < 0.05 vs. CCI + control group.

To investigate the interaction between PPAR γ and M1-phenotype microglial activation in the spinal cord, the expression of microglial specific protein Integrin αM, M1 marker (CD86), and proinflammatory signaling molecules (CX3CR1/p38MAPK and ERK) was assessed in rats. Figure 2 showed that compared to the sham group, the levels of Integrin αM, CX3CR1, p-p38 MAPK, and p-ERK proteins and CD86 were increased in the CCI group on POD 7. Compared with the CCI group, the expression of the molecules above was decreased in the CCI + PPAR γ group (*p* < 0.05). The results showed that PPAR γ inhibited the activation of M1 microglia and CX3CR1, p38MAPK, and ERK signaling pathways in the spinal cord of CCI rats. The expression of anti-inflammatory cytokine IL-10 was also measured. Figure 2B showed that compared to the CCI group, IL-10 protein levels were increased in the CCI + PPAR γ group on POD 7 (*p* < 0.05). The results suggested that PPAR γ overexpression increased IL-10 production in the spinal cord of CCI rats.

### PPAR γ Overexpression in BV-2 Microglia

**FIGURE 3:**
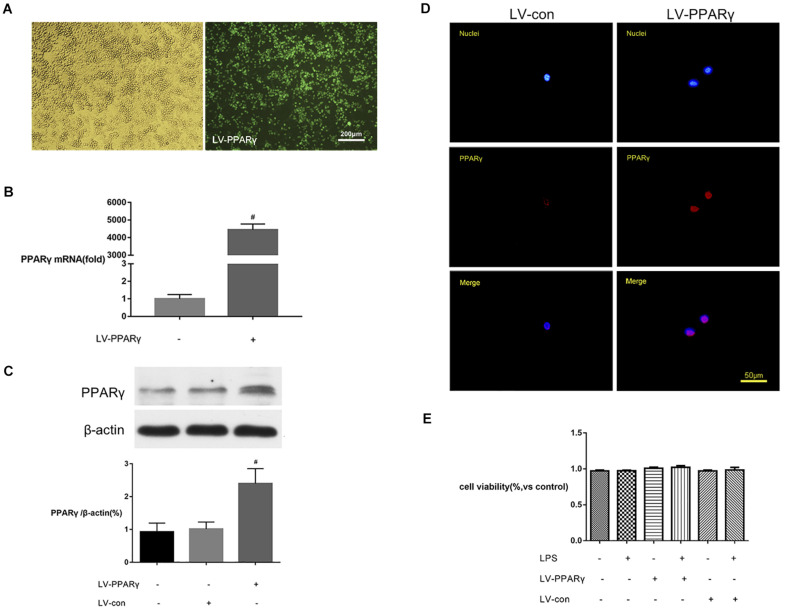
PPAR γ overexpression in BV-2 microglial cells. **(A)** The transfection efficiency was estimated with enhanced GFP by microscopy. Comparison of cells in the bright field (left) and fluorescence field (right) suggested a transfection efficiency of more than 90%. **(B,C)** Real-time qPCR and Western blotting analysis showed that the levels of PPAR γ mRNA **(B)** and protein **(C)** were increased in LV-PPAR γ overexpressed cells compared to the control cells. **(D)** Immunocytochemistry analysis showed that the increased PPAR γ expression was mainly distributed in the nuclei. Nuclei were labeled with DAPI (blue) and PPAR γ was labeled with antibody (red). **(E)** CCK-8 assay showed that LV-PPAR γ transfection and LPS (1 μg/mL) treatment had no effects on the viability of BV-2 cells. The results are expressed as mean ± SEM, *n* = 4. ^#^*P* < 0.05 vs. LV-control group.

To further explore the effects of PPAR γ on microglial activation, BV-2 microglial cells were transfected by LV-PPAR γ. To confirm the overexpression of PPAR γ in BV-2 cells, the expression of labeled GFP in cells was evaluated by fluorescence microscopy. Figure 3A showed that cell bodies were highly fluorescent after transfection of the lentiviral vector. Figures 3B,C showed that the mRNA and protein overexpression of PPAR γ was further confirmed by real-time qPCR and Western blot. Figure 3D showed that immunocytochemistry analysis revealed that the increased PPAR γ protein was mainly distributed in the nuclei. The results indicated that the transfection efficiency was sufficient for further experiments.

To demonstrate whether the overexpression of PPAR γ was toxic to microglial growth, the viability of BV-2 microglial cells in different experimental groups was tested by CCK-8 assay. Figure 3E showed that based on the CCK-8 assay, the values of cell viability were not significantly different between groups, indicating that lentivirus-mediated PPAR γ overexpression did not affect the viability of BV-2 microglia.

### Effects of PPAR γ Overexpression on M1 Activation of BV-2 Microglia

**FIGURE 4:**
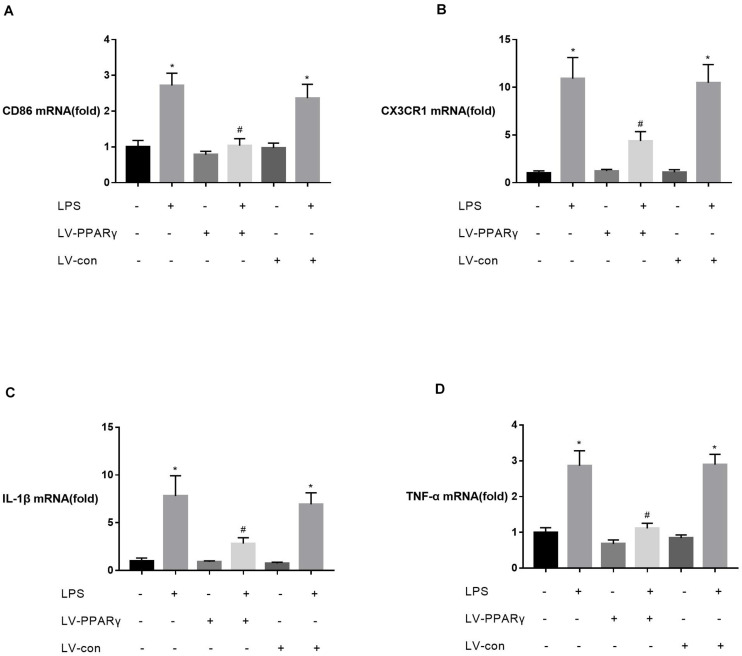
Effects of PPAR γ overexpression on the levels of CD86 **(A)**, IL-1β **(C)**, TNF-α **(D)**, and CX3CR1 **(B)** mRNA in BV-2 cells upon stimulation with LPS (1 μg/mL). **(A–D)** Real-time qPCR showed that the levels of CD86, CX3CR1, IL-1β, and TNF-α mRNA after 2 h of stimulation were significantly attenuated in PPAR γ overexpressed cells compared to the control cells. Data are expressed as mean ± SEM, *n* = 4. *P < 0.05 vs. sham group and ^#^*P* < 0.05 vs. LV-control group.

To investigate the relationship between PPAR γ expression and M1 activation of microglia, the expressions of M1 marker CD86, M1-type cytokines (IL-1β and TNF-α), and CX3CR1 were assessed *in vitro* in BV-2 microglia. Figures 4A–D showed that the basic mRNA levels of CD86 **(A)**, CX3CR1 **(B)**, IL-1β **(C)**, and TNF-α **(D)** were not significantly different between PPAR γ-overexpressed cells and the control cells. The mRNA expressions of CD86 **(A)**, CX3CR1 **(B)**, IL-1β **(C)**, and TNF-α **(D)** were significantly increased after 2 h of BV-2 microglia incubation with LPS. Compared to the control cells, the mRNA levels of CD86 **(A)**, CX3CR1 **(B)**, IL-1β **(C)**, and TNF-α **(D)** were significantly decreased in PPAR γ-overexpressed cells (*P* < 0.05), indicating that PPAR γ overexpression inhibited M1 activation in BV-2 microglia *in vitro*.

**FIGURE 5:**
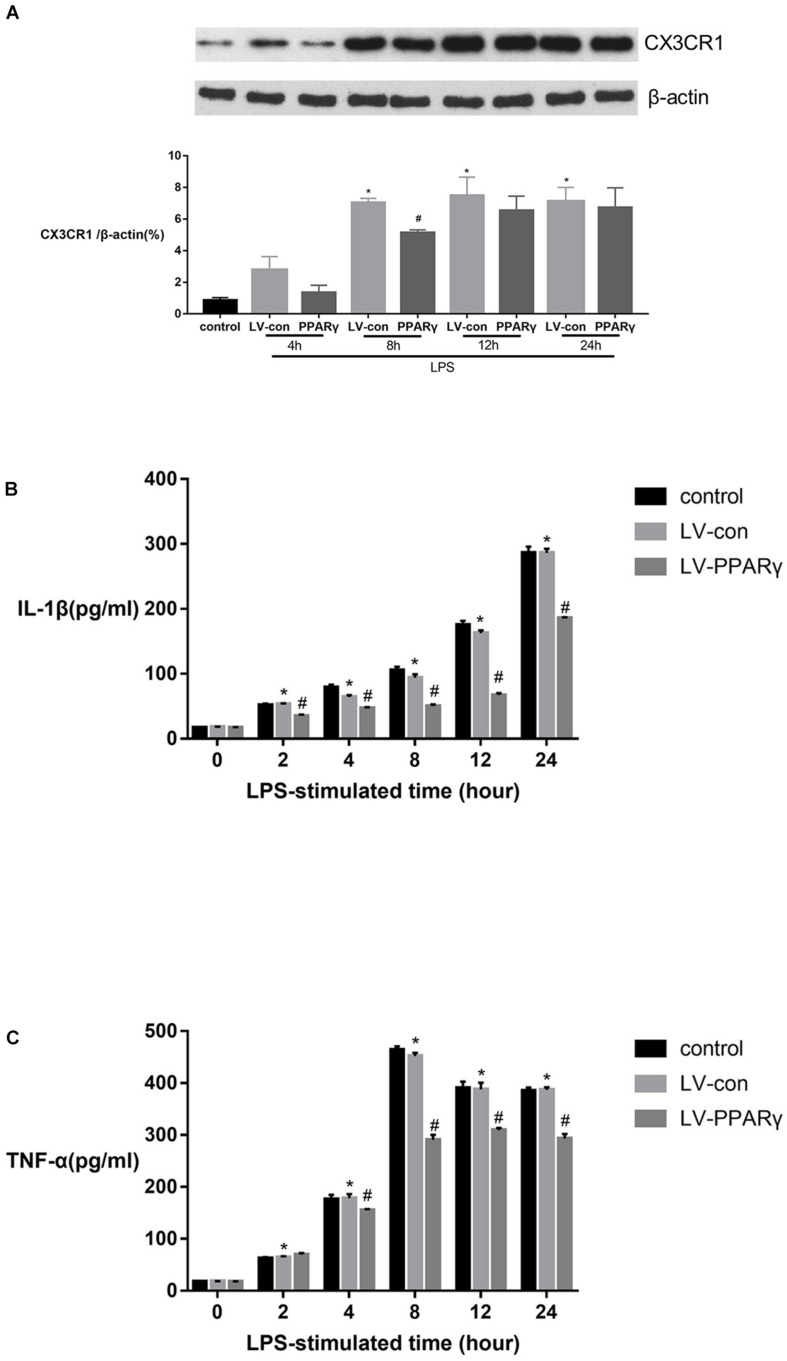
Effects of PPAR γ overexpression on the protein levels of IL-1β **(B)**, TNF-α **(C)** and CX3CR1 **(A)** in BV-2 cells stimulated by LPS (1 μg/mL). **(A)** As shown by Western blotting, the expression of CX3CR1 protein was significantly decreased in PPAR γ overexpressed cells compared to the control cells after 8 h of stimulation. **(B,C)** As demonstrated by ELISA, the secretions of IL-1β and TNF-α were significantly decreased in the supernatant of PPAR γ overexpressed cells compared to control cells after 8, 12, and 24 h of stimulation by LPS. Data are expressed as mean ± SEM, *n* = 4. *P < 0.05 vs. sham group and ^#^*P* < 0.05 vs. LV-control group.

Figure 5A showed that the levels of CX3CR1 protein were increased in BV-2 microglia after 8, 12, and 24 h of stimulation with LPS (*p* < 0.05). Compared to the control cells, the up-regulated levels of CX3CR1 protein were significantly decreased in PPAR γ-overexpressed cells after 8 h of LPS stimulation (*p* < 0.05). The results indicated that PPAR γ overexpression inhibited LPS-induced CX3CR1 protein upregulation in BV-2 microglia.

Figures 5B,C showed that based on ELISA assay, the expression of IL-1β and TNF-α was significantly increased in the supernatant of cell culture after 2 h of LPS stimulation and peaked at 24 and 8 h, respectively. The productions of IL-1β and TNF-α were significantly reduced in the supernatant layer of PPAR γ overexpressed cells at 4, 8, 12, and 24 h (*p* < 0.05). These results revealed that PPAR γ overexpression inhibited excessive productions of IL-1β and TNF-α in microglia challenged with LPS.

## Discussion

There is abundant evidence indicating that neuroinflammation induced by nerve injury may be a main cause for NP (Ji et al., 2018; Matsuda et al., 2019). Nerve injury initiates activation of microglia and promotes pro-inflammatory responses in the nervous system, thereby compromising the normal microglia-neuron communication and contributing to central sensitization. In pre-clinical studies, PPAR γ is well known for its analgesic effect, but its analgesic mechanism remains elusive (Freitag and Miller, 2014; Jia et al., 2016). To investigate the effects of PPAR γ on neuroinflammation and microglia activation in the spinal cord, sciatic CCI was used to establish the NP model. Consistent with previous studies, injury over 7 days induced lower pain thresholds to mechanical stimuli and radiant heat at the ipsilateral hind paw of the injured rats compared to the untreated ones. This indicates the developed NP behaviors, thus suggesting the validity of the NP model used in the study.

The role of microglia in NP has recently caught attention. Previous studies have indicated that activation of microglia in the spinal cord is involved in the onset and progression of NP (Calvo and Bennett, 2012; Chen et al., 2018; Shen et al., 2019). Microglia are the first line of the innate immune system in the CNS and respond quickly to nerve injury. Studies have proved that a change in polarity of spinal microglia acts as a trigger for persistent neuroinflammation and NP (Xu et al., 2016). Nerve injury can prime microglia toward the M1 phenotype, which is responsible for provoking neuroinflammation. This study found that CCI rats had a higher level of M1 marker CD86, along with a low level of M2 marker IL-10 in the spinal cord of CCI rats. CD86 is known as a surface marker for M1 microglia and provides costimulatory signals necessary for T lymphocyte activation. As IL-10 is associated with the suppression of inflammation, promotion of tissue repair and reversal of NP, the imbalance of M1/M2 phenotype is implicated in NP pathogenesis, which is consistent with the previous report (Xu et al., 2016). The increased level of microglia marker Integrin αM, accompanied by increased expression of CX3CR1 as well as phosphorylated p38MAPK and ERK, indicated CCI-induced spinal microgliosis. The increased levels of CX3CR1 and phosphorylated p38MAPK and ERK in spinal microglia may lead to the activation of M1-type intracellular signaling pathways and excessive production of pro-inflammatory cytokines, which prolong the neuroinflammation and result in NP progression (Wang A. et al., 2018; Zhong et al., 2018).

PPAR γ is a member of PPARs, a group of nuclear hormone transcription factors. The presence of PPAR γ at key sites involved in pain procession, such as the spinal cord, has been demonstrated and PPAR γ has emerged as the main modulator in pain processing. In this study, the analgesic effects of lentivirus encoding PPAR γ, upon intrathecal administration, indicate its modulatory role in spinal pain processing in the rat model of CCI. The data suggested that PPAR γ acts as a key inhibitor of spinal M1 microglia activation. The lentivirus-mediated PPAR γ overexpression suppressed the spinal M1-type marker CD86, microglial CX3CR1, intracellular signaling of phosphorylated p38MAPK and ERK. Meanwhile, the spinal expression of IL-10 was significantly increased in NP rats. However, the overexpression of PPAR γ did not change the pain behaviors in sham operated animals, indicating that the up-regulated PPAR γ itself has no effects on the inactivated microglia. Previous studies showed that intrathecal administration of recombinant IL-10 was anti-allodynic by modulating microglial activation in the mouse model of acute stage of complex regional pain syndrome (Kim et al., 2018). Overall, the data from this study indicate that PPAR γ may reverse neuroinflammation by increasing the levels of anti-inflammatory factors and reducing the levels of pro-inflammatory factors in the spinal cord, thereby playing a protective role against NP. Thus, the lentivirus-mediated PPAR γ overexpression may provide a feasible method to reverse the spinal pathogenesis of NP. However, further experiments are needed to clarify the exact mechanisms of these molecules.

Further *in vitro* experiments were carried out to investigate the effects of PPAR γ on M1 microglia phenotype and CX3CR1 expression. Based on the results, PPAR γ overexpression suppressed LPS-induced CX3CR1 expression and M1-type activation in BV-2 microglia. PPAR γ overexpression decreased the levels of CD86, IL-1β and TNF-α, but had no effects on the levels of these mediators in untreated BV-2 microglia. It was suggested that the M1 phenotype and receptor expression of microglia could be directly modulated by the level of PPAR γ and was dependent on the functional state of the cell. Higher levels of PPAR γ can reduce the expression of proinflammatory mediators, and help curb the progression of neuroinflammation and maintain the homeostasis of the nervous system. Interestingly, the level of PPAR γ may be regulated by other mediators (Mizutani et al., 2007). Previously, it was shown that a lower level of CX3CR1 expression can increase PPAR γ expression and inhibit inflammation of intestinal macrophages (Malur et al., 2009). Even though the role of post-transcriptional mechanism cannot be ruled out, the data from this study showed that PPAR γ regulated CX3CR1, IL-1β, and TNF-α expression mainly at the transcriptional and translational levels. Some studies have proven that PPAR γ can be directed to a specific nuclear co-repressor/histone deacetylase three complex bound to the promoter regions of inflammatory genes, thus keeping the target gene inactive (Wen et al., 2010). PPAR γ also acts in a non-genomic manner to suppress nuclear factor-κ B, STAT-1, and AP-1 signaling pathways (Moraes et al., 2006). Further research is needed to reveal the exact mechanism of PPAR γ responsible for CX3CR1 expression and microglia function.

Although it was found that PPAR γ overexpression did not influence the cellular growth under the experimental conditions utilized here and the results suggested that the therapy of PPAR γ overexpression in spinal tissues may be safe in clinical settings, further *in vivo* neurotoxicity studies are needed.

There are still some limitations in this study. Firstly, we only studied the analgesic effect of intrathecal injection of LV-PPAR γ on the 7th day of CCI and further studies are needed to illuminate the duration and mechanism of the analgesic effect of PPAR γ overexpression. Secondly, a previous study demonstrated that antagonizing PPAR γ also promoted the M1-to-M2 shift in microglia *in vitro*, which might be due to the activation of the LKB1–AMPK signaling pathway (Ji et al., 2016). Further research is needed to clarify the regulatory mechanisms between CD86 and other surface markers of microglial phenotypes and the PPAR γ pathway.

In conclusion, this study showed that overexpression of PPAR γ can prevent the development of NP by inhibiting M1 microglia activation, CX3CR1 expression, and activation of p38MAPK and ERK signaling while increasing IL-10 expression in the spinal cord of CCI rats. To the knowledge of the authors, this study is the first to show the inhibitory effects of PPAR γ on the expression of CD86, CX3CR1, and phosphorylated p38MAPK in the spinal tissue. *In vitro* experiments further confirmed the inhibitory effects of PPAR γ on the M1 phenotype and CX3CR1 expression of microglia. The authors have reported a novel mechanism for PPAR γ to regulate neuroinflammation, and intrathecal infusion of lentivirus encoding PPAR γ may be a promising candidate for clinical treatment of NP.

## Data Availability Statement

The raw data supporting the conclusions of this article will be made available by the authors, without undue reservation.

## Ethics Statement

The animal study was reviewed and approved by Institutional Animal Care and Use Committee of Central South University.

## Author Contributions

XL helped with cells culture, lentivirus transfection, western blot and real-time polymerase chain reaction experiments, analyzed the data, and wrote the manuscript. QG was involved in study design and revising the manuscript. ZY helped with cells culture and analyzed the data. EW helped with western blot technique and supervised the study. WZ helped with enzyme-linked immunosobent technique. ZS helped with real-time polymerase chain reaction technique. ZH helped with recombinant lentivirus technique. TZ was involved in study design. YW helped statistical analysis. YP helped conduct the study, analyzed the data, and wrote the manuscript. All authors approved the final version of the manuscript.

## Conflict of Interest

The authors declare that the research was conducted in the absence of any commercial or financial relationships that could be construed as a potential conflict of interest.

## Abbreviations

NP: neuropathic pain
LV-PPAR γ: lentivirus encoding PPAR γ
CNS: central nervous system
EGFP: enhanced green fluorescence protein
MWT: mechanical withdrawal threshold
PWT: Paw withdrawal threshold
PWL: Paw withdrawal latency
RT: room temperature
DAPI: 4′,6-diamidino-2-phenylindole
POD: postoperative day.
